# Domestication shapes the gut microbial structure and metabolic function in felids: a metagenomic study of wild and domestic cats

**DOI:** 10.3389/fmicb.2026.1828152

**Published:** 2026-07-28

**Authors:** Jinfa Chen, Wenlu Fan, Xuewei Chen, Han Zhang, Miao Feng, Shunfu He, Changjun Song, Jialiang Wang

**Affiliations:** 1 Pet Nutrition Research Center, Gambol Pet Food Group Co., Ltd., Shanghai, China; 2 Wildlife Rescue and Breeding Center, Qinghai, China

**Keywords:** domestication, gut microbiota, wild felids, domestic cats, metagenomics, metabolic pathways

## Abstract

**Introduction:**

The domestication process has profoundly altered the dietary patterns and living conditions of cats, with corresponding effects on their gut microbiome.

**Methods:**

This study compared the gut microbiota composition and metabolic functions between wild felids (*Otocolobus manul* and *Felis bieti*) and domestic cats using metagenomic sequencing.

**Results:**

Taxonomic analysis revealed significantly higher microbial alpha diversity and distinct community structure in wild felids compared to domestic cats. The gut microbiota of domestic cats was characterized by a higher relative abundance of Bacteroidota (when compared to *F. bieti*) and of Pseudomonadota, Uroviricota, and Cyanobacteriota, as well as an enrichment of carbohydrate-associated genera such as *Segatella*. In contrast, wild felids exhibited enrichment of potential pathogens (e.g., *Clostridium perfringens, Escherichia coli*) and genera including *Clostridium* and *Fusobacterium*, alongside a higher abundance of microbial genes linked to protein degradation and fermentation. Functional metagenomic analysis further identified consistent differences in microbial metabolic potential across both wild species comparisons. Wild felids showed higher abundances of genes involved in butyrate production, lysine degradation, and de novo synthesis of vitamins and cofactors. Domestic cats, in contrast, exhibited enrichment of genes for plant polysaccharide hydrolysis, ketone body formation, aromatic amino acid biosynthesis, and salvage of folate derivatives.

**Discussion:**

These results suggest that domestication is associated with a shift in the gut microbial functional repertoire - from a butyrogenic, protein-catabolic, and de novo-synthesizing profile in wild felids toward a more carbohydrate-hydrolyzing, ketogenic, and salvage-oriented profile in domestic cats, reflecting dietary and environmental adaptations.

## 1 Introduction

As important companion animals to humans, cats have a large global captive population. The domestication history of the domestic cat can be traced back to approximately 10,000 years ago in the Fertile Crescent region (Baca et al., 2018), with the *Felis silvestris lybica* as its direct ancestor (Nilson et al., 2022). The proliferation of domestic cats as pets, driven by the acceleration of urbanization and lifestyle changes, has led to a concomitant increase in the demand for health management and nutritional interventions. In contrast to omnivorous animals such as humans or dogs, felines within the Carnivora order have a distinctive carnivorous dietary structure that endows their gut microbiota with characteristic carnivore attributes (Moon et al., 2018), rendering them an optimal model for investigating host-microbiome coevolution.

In recent years, research on the feline gut microbiome has increased, revealing significant associations with host health. Previous studies have shown that the gut microbiota of cats consists of the core phyla Bacillota, Bacteroidota, Fusobacteriota, Pseudomonadota, and Actinomycetota (Li et al., 2022; Páez-Triana et al., 2025). At the genus level, the most abundant core genera included *Segatella, Bacteroides, Collinsella, Blautia*, and *Megasphaera* (Ganz et al., 2022). Current research indicates that significant behavioral and ecological divergence exists between the domestic cat (*Felis silvestris catus*) and its wild relatives, which may be mediated through hormonal regulation and alterations in the gut microbiota (Chong et al., 2022). Notably, the morphological and genetic changes in domestic cats resulting from the domestication process may be associated with structural changes in their gut microbial communities. This suggests that the gut microbiota of cats is closely associated with their genetic background, dietary structure, and living environment.

While domestication inherently involves profound shifts in diet and living environment, disentangling their individual effects on the gut microbiome is challenging. In this study, by comparing wild felids consuming a natural raw diet in a complex environment with domestic cats consuming a processed commercial diet in a controlled setting, we aim to capture the integrated impact of the domestication process on the feline gut microbiome.

## 2 Materials and methods

### 2.1 Experimental animals and sample collection

This study included 20 animals. The wild group consisted of four Pallas's cats (*Otocolobus manul*) and six Chinese mountain cats (*Felis bieti*), obtained from the Qinghai-Tibet Plateau Wildlife Park. These animals were fed an untreated raw meat-based diet consisting of raw mutton, chicken, and whole pigeons provided every other day. The domestic group consisted of five British Shorthair and five Ragdoll cats from the Nutrition Research and Development Center of Gambol Pet Group Co., Ltd., and were fed measured amounts of a commercial extruded diet at scheduled times twice daily. Since the wild felines were rescued rather than bred, their exact ages cannot be determined. However, it is known that they are all adults. The animal basic metadata is shown in Supplementary Table S1. Both wild and domestic animals lived in pairs in a free activity area of approximately 50 square meters. All animals had reached adulthood and were confirmed to be healthy, with no gastrointestinal or excretory disorders observed during a 1-month pre-test period, and with no administration of any drugs or probiotics for at least 6 months prior to inclusion in the study. All fecal samples were obtained via natural defecation. Professional personnel aseptically collected samples from the internal mid-section of the feces to avoid contamination. All samples were processed within 3 h of defecation and stored at −80 °C.

### 2.2 Fecal DNA extraction and metagenomic sequencing

Genomic DNA was extracted from fecal samples using the MagPure Stool DNA KF Kit B (Magen, Guangzhou, China) according to the manufacturer's protocol. Briefly, 100–200 mg of each sample was transferred to a tube containing grinding beads. Then, 1 mL of Buffer ATL/PVP-10 was added, and the sample was homogenized using a grinding machine (Shanghai Jingxin Tech, China) followed by incubation at 65 °C for 20 min. After centrifugation at 14,000 × g for 5 min (Eppendorf, Germany), the supernatant was transferred to a new tube. Subsequently, 0.6 mL of Buffer PCI was added, and the mixture was vortexed thoroughly for 15 s. Following centrifugation at 18,213 × g for 10 min, the supernatant was subjected to automated nucleic acid purification using a Kingfisher system (Thermo Fisher, USA) with magnetic beads. The purification steps (binding, washing, and elution) were performed according to the kit's instructions. Finally, the eluted DNA was collected in a 1.5 mL tube.

The purified DNA was fragmented to an average size of 200–400 bp using a Covaris ultrasonicator. The fragments were size-selected with magnetic beads. A sequencing library was then constructed through end repair, A-tailing, adapter ligation, and PCR amplification. The PCR product was purified, denatured, and circularized to generate single-stranded circular DNA. Finally, sequencing was performed on an MGISEQ 2000 platform (PE150). In this process, the circular DNA was amplified by rolling circle replication to produce DNA nanoballs (DNBs), which were loaded into a patterned nanoarray chip and sequenced using combinatorial probe-anchor synthesis (cPAS).

Raw sequencing reads were processed for quality control and filtering using SOAPnuke (v 2.3) with the following criteria: 1) removal of reads containing ≥10% ambiguous bases (N); 2) removal of reads with adapter contamination (allowing ≥15 bp aligned to adapter sequences); and 3) removal of reads with >50% low-quality bases (Phred score < 20). The retained reads were then aligned to the host genome (*Felis catus* assembly felCat9) using Bowtie2 (v 2.4.4), and host-derived sequences were removed. The resulting clean data comprised an average of (36.3 ± 7.2) million reads per sample (mean ± standard deviation).

### 2.3 Taxonomic and functional annotation

Taxonomic assignment was performed directly on the clean reads using Kraken2 (v 2.1.2) with default parameters, against a custom database constructed from the NCBI NT subset (k2_pluspf_16gb_20240112). Species-level abundances were then estimated using Bracken (v2.6.1). To further assess taxonomic composition and to estimate the proportion of reads assignable to known vs. unknown lineages, we additionally performed read-based profiling using MetaPhlAn 4 (v4.0.1) with the ChocoPhlAn database (mpa_vOct22_CHOCOPhlAnSGB_202403).

As for functional annotation, clean reads were assembled with MEGAHIT (v 1.2.9) (k-mer-based), and contigs ≥200 bp were retained for downstream analysis. Genes were predicted *de novo* using MetaGeneMark (v 3.38), followed by dereplication with CD-HIT (v 4.8.1). Gene abundance was quantified (TPM) using Salmon (v 1.6.0). Functional annotation of the non-redundant genes was performed via BLASTP using DIAMOND (v 0.8.24) against the KEGG database (v 101.0) and the CAZy database (v 20240326). The DIAMOND parameters were: –evalue 1e-5 –threads 5 –outfmt 6 –seg no –max-target-seqs 20 –more-sensitive -b 0.5 –salltitles. The gene catalog statistics, including total predicted genes, number of non-redundant genes were summarized in Supplementary Table S2.

The relative abundance of each functional category (KEGG ortholog [KO] or pathway level 3) was calculated as the sum of the relative abundances of all genes annotated to that category. For KEGG level-3 pathways, the abundances of all KOs assigned to a given pathway were directly summed.

### 2.4 Statistical analysis

Statistical analyses and visualizations were performed using R software (v4.5.0) and the BGI Biosystem cloud platform (https://biosys.bgi.com/). Rarefaction curves were generated to confirm that Chao1 diversity estimates had reached saturation, and diversity indices were calculated after rarefying to a common sequencing depth. Differences in microbial community composition were assessed using PERMANOVA (adonis2 in the vegan package with 999 permutations; *R*^2^ values are reported), Wilcoxon rank-sum tests, fold change analysis, and linear discriminant analysis effect size (LEfSe). All *p*-values were corrected using the Benjamini-Hochberg false discovery rate (FDR) method. For LEfSe, the LDA score threshold was set to >3, and the Kruskal-Wallis test *p*-value threshold was < 0.05. Pathway-level differences were evaluated using the Reporter Score method (Patil and Nielsen, 2005): for each KO, a Wilcoxon rank-sum test *p*-value was transformed into a *Z*-score; the pathway *Z*-score was calculated as the mean *Z* of its constituent KOs, normalized by the standard deviation of permuted values (1,000 permutations), and a corrected *Z*-score >1.65 or < -1.65 was considered significant (*p* < 0.05), using the entire annotated gene catalog as the background set. Statistical significance levels in figures are indicated as follows: ^*^FDR < 0.05, ^**^FDR < 0.01, and ^***^FDR < 0.001.

## 3 Results

### 3.1 Sequencing depth and taxonomic classification efficiency

Rarefaction curves approached saturation for all samples (Supplementary Figure S1), indicating that the sequencing depth was sufficient to capture the majority of gut microbial diversity in both domestic and wild felids. Sequencing quality metrics, including the percentage of reads passing QC and the percentage of host reads removed for each sample, were summarized in Supplementary Table S3. Taxonomic classification rates (proportion of reads assigned to any known taxon) were assessed using two independent methods. With Kraken2 against the NCBI NT database, the mean classification rate was significantly higher in domestic cats (45.2 ± 7.5%) than in wild felids (28.2 ± 3.9%; *p* < 0.001, Welch's *t*-test). Using MetaPhlAn 4, which incorporates a larger set of marker genes including unknown species-level genome bins (uSGBs), classification rates were substantially improved in both groups, yet remained significantly higher in domestic cats (75.3 ± 3.2%) compared to wild felids (61.3 ± 8.7%; *p* < 0.001, Welch's *t*-test). Notably, the uSGBs fraction—reads assigned to uncharacterised microbial lineages—accounted for a substantial proportion of the total reads in both groups, averaging 37.43 ± 6.52% in domestic cats and 38.87 ± 10.01% in wild felids, with no significant difference between groups (*p* > 0.05, Welch's *t*-test). This indicates that a considerable portion of the feline gut microbiome remains taxonomically unexplored, even when leveraging an expanded reference database. The consistently lower overall classification rates in wild samples likely reflected the greater phylogenetic divergence of their gut microbiota from currently available reference genomes. Assembly statistics and read mapping rates for each sample were provided in Supplementary Table S4. Detailed classification statistics, including known and unknown SGBs proportions for each sample, were provided in Supplementary Table S5. To ensure consistency in downstream analyses, all subsequent taxonomic analyses were performed using the Kraken2-based classification results.

### 3.2 Diversity and composition of the gut microbiota

**Figure 1:**
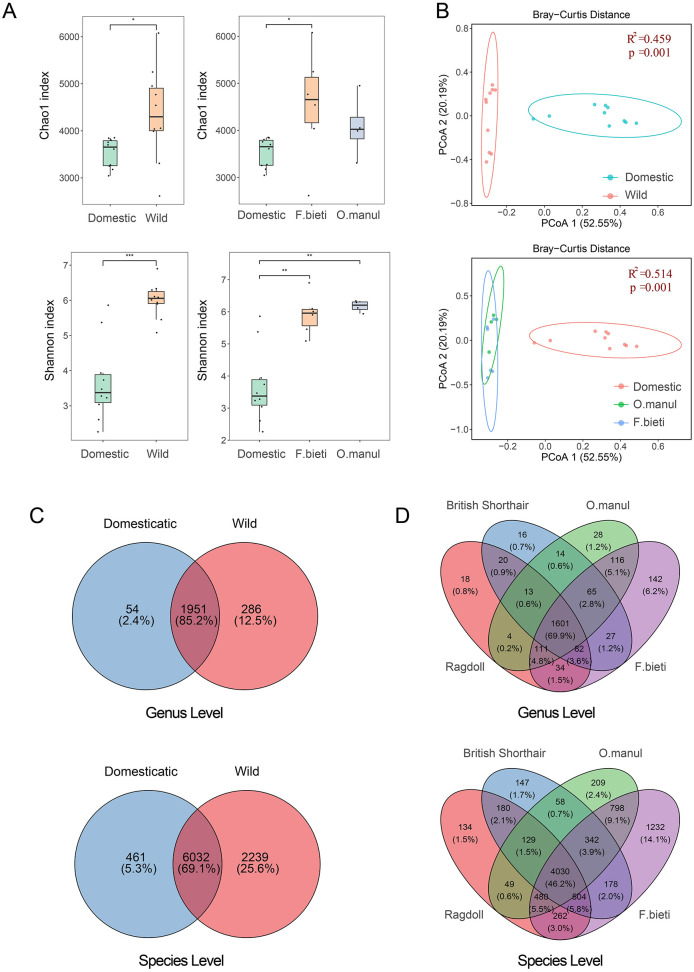
Diversity and Composition of the Gut Microbiota. **(A)** Boxplot of the Chao1 index and Shannon index. **(B)** PCoA plot of β-diversity based on Bray-Curtis dissimilarity. **(C)** Venn plot at the genus and species levels between the wild group and the domestic group; **(D)** Venn plot at the genus and species levels among the four breed groups.

Alpha diversity was compared using the Chao1 and Shannon indices. As shown in Figure 1A, the combined wild group (including both *O. manul* and *F. bieti*) exhibited significantly higher gut microbial alpha diversity than the domestic group (*p* < 0.05). When the two wild species were analyzed separately, *F. bieti* showed significantly higher Chao1 and Shannon indices compared to domestic cats (*p* < 0.05). *O. manul* also exhibited a higher Shannon index than domestic cats (*p* < 0.05), but the difference in Chao1 index did not reach statistical significance (*p* = 0.056), possibly due to the small sample size (*n* = 4). No significant differences in either alpha diversity index were detected between the two wild species.

Beta diversity assessed by Bray-Curtis dissimilarity and visualized via principal coordinate analysis (PCoA) revealed that both *O. manul* and *F. bieti* differed significantly from domestic cats in overall microbial community composition, whereas no significant difference was detected between the two wild species (Figure 1B; *p* < 0.05, PERMANOVA test). Additionally, comparison of beta diversity among the four groups (two domestic breeds and two wild species) confirmed no significant differences between the two domestic breeds and no significant differences between the two wild species, further supporting the validity of the grouping strategy (Supplementary Figure S2).

Venn diagram analysis revealed that at the genus level, the domestic group harbored 54 unique genera, while the wild group possessed a substantially larger set of 286 unique genera. At the species level, this disparity was even more pronounced, with 461 species unique to the domestic group and 2,239 species unique to the wild group (Figure 1C; permutation test, FDR < 0.01). We further split the samples into four subgroups (British Shorthair, Ragdoll, *F. bieti*, and *O. manul*), thereby achieving more balanced group sizes for comparison. As shown in Figure 1D; *F. bieti* exhibited a greater number of unique genera and species compared to each of the two domestic breeds (permutation test, FDR < 0.05), whereas *O. manul* did not show significant differences (FDR > 0.05), likely due to its limited sample size. Detailed permutation test results are provided in Supplementary Table S6.

**Figure 2:**
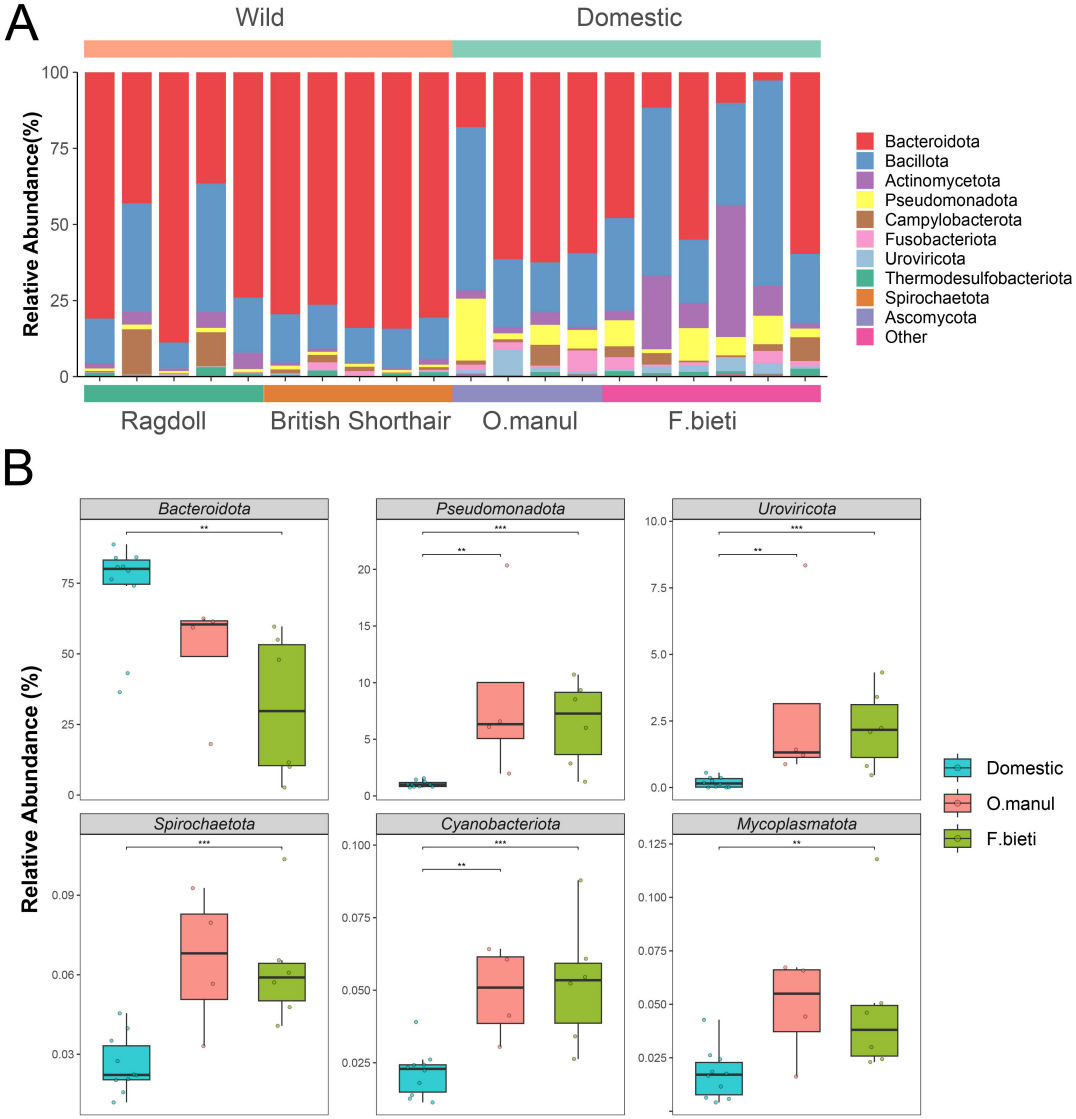
Comparison of differences at the phylum level of gut microbiota. **(A)** Stacked bar plots of gut microbiota at the phylum level. **(B)** Boxplot of the relative abundance of bacterial phyla that differed significantly between groups.

The stacked bar chart showed the relative abundance of the gut microbiota at the phylum level in all samples of each group (Figure 2A). Bacteroidota and Bacillota were identified as the most abundant phyla in all groups. We then compared the differences between domestic cats, *F. bieti*, and *O. manul*. Six phyla with the highest relative abundances and significant differences among groups were identified (Figure 2B). The relative abundance of Bacteroidota was significantly higher in the domestic group than in the *F. bieti* group. Conversely, the domestic cats exhibited significantly lower relative abundances of Spirochaetota and Mycoplasmatota compared to the *F. bieti* group, whereas no significant differences in these two phyla were observed between the domestic group and the *O. manul* group. Furthermore, the relative abundances of Pseudomonadota, Uroviricota, and Cyanobacteriota were significantly higher in the domestic group than in both wild felid groups (*F. bieti* and *O. manul*) (FDR < 0.05).

### 3.3 Identification of differential gut microbes

**Figure 3:**
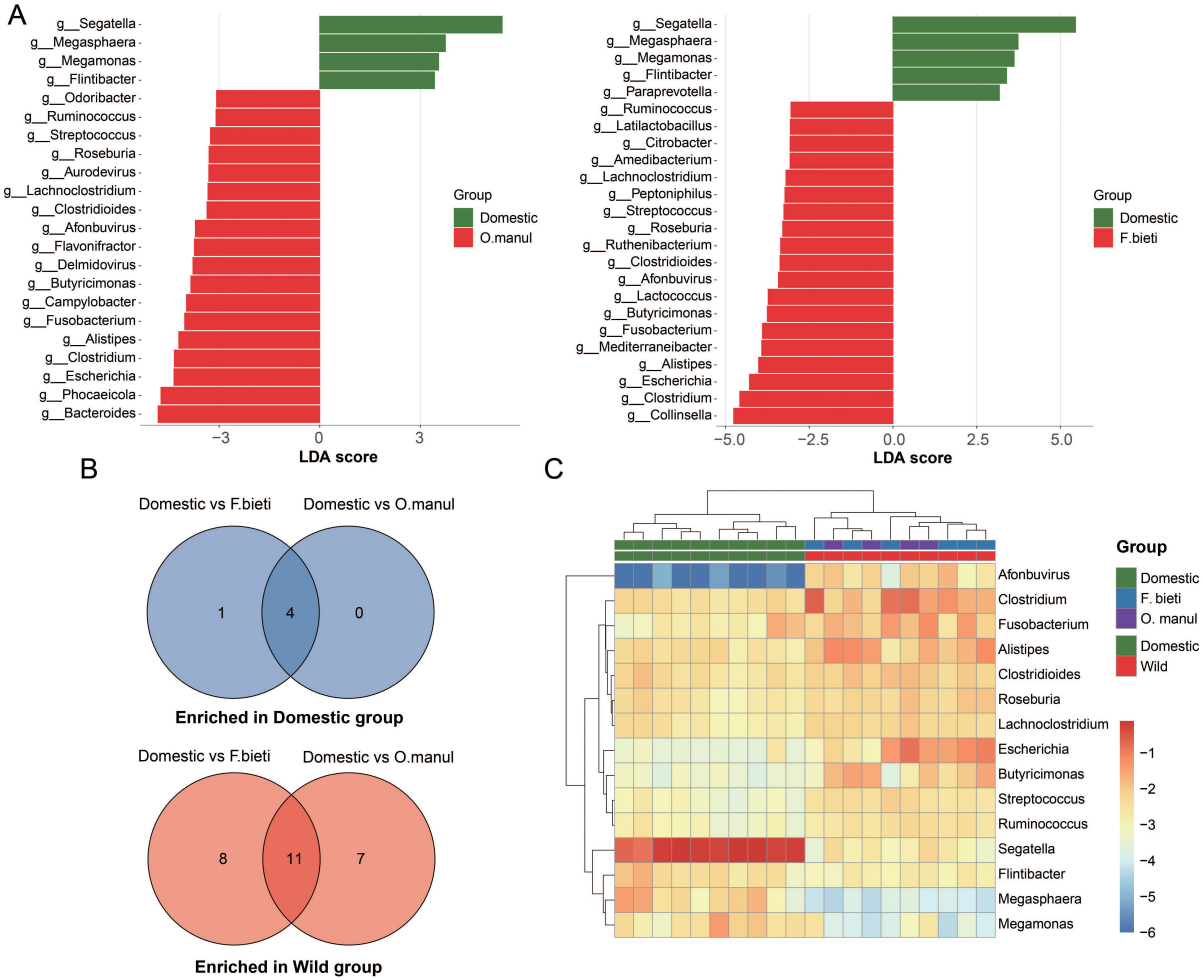
Comparison of differences at the genus level of gut microbiota. **(A)** Bar plot of linear discriminant analysis (LDA) scores. **(B)** Venn diagram of LDA results. **(C)** Heatmap of the relative abundances of the 15 consistent genera across individual samples.

Differential genera between domestic cats and each wild felid were identified using LEfSe analysis (Kruskal–Wallis test, *p* < 0.05; linear discriminant analysis (LDA) score > 3). As shown in Figure 3A, comparison between domestic cats and *O. manul* revealed that four genera were enriched in the domestic group: *Segatella, Megasphaera, Megamonas*, and *Flintibacter*. In contrast, 18 genera were significantly more abundant in *O. manul*, including *Bacteroides, Phocaeicola, Escherichia, Clostridium, Alistipes*, and *Fusobacterium*. Similarly, in the comparison between domestic cats and *F. bieti*, five genera were overrepresented in domestic cats: *Segatella, Megasphaera, Megamonas, Flintibacter*, and *Paraprevotella*. The *F. bieti* group harbored 19 enriched genera, such as *Collinsella, Clostridium, Escherichia, Alistipes, Mediterraneibacter*, and *Fusobacterium*. Complete LEfSe bar plots showing all taxonomic levels and the corresponding cladograms for both comparisons are provided (Supplementary Figures S3, S4).

To identify genera consistently altered across both comparisons, we intersected the two sets of differentially abundant genera. Venn diagram analysis (Figure 3B) showed that four genera were consistently enriched in domestic cats (*Segatella, Flintibacter, Megasphaera, Megamonas*), while 11 genera were consistently enriched in both wild felids (*Afonbuvirus, Clostridium, Fusobacterium, Alistipes, Clostridioides, Roseburia, Lachnoclostridium, Escherichia, Butyricimonas, Streptococcus, Ruminococcus*). A heatmap was generated to visualize the abundance patterns of these 15 consistent genera across individual samples (Figure 3C). The clustering clearly separated wild from domestic samples, confirming the robustness of the identified signatures.

**Figure 4:**
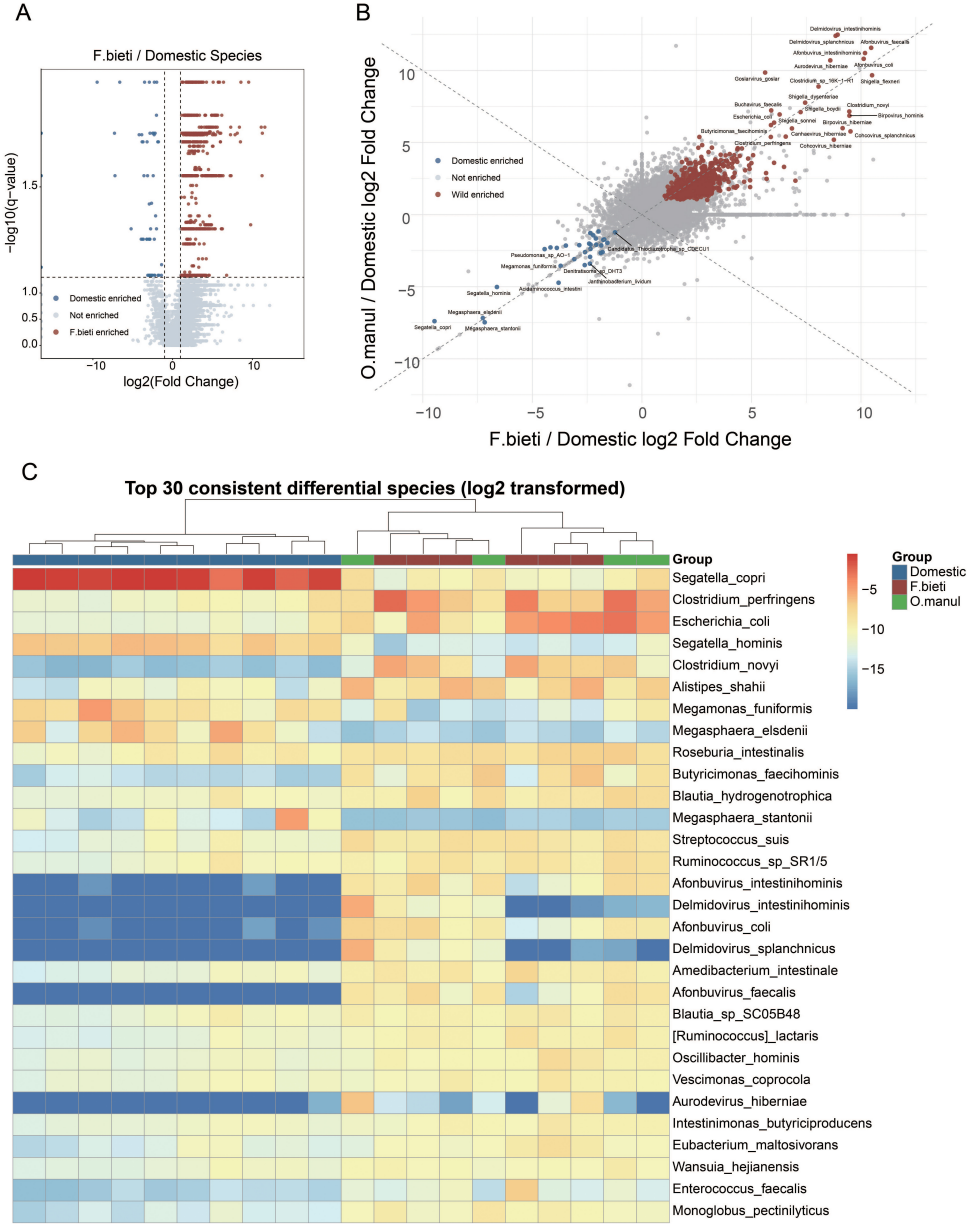
Comparison of differences at the species level of gut microbiota. **(A)** Volcano plot of differentially abundant species between domestic cats and *F. bieti*. **(B)** Scatter plot showing consistency of fold changes between the two comparisons. Red: consistently enriched in wild felids; blue: consistently enriched in domestic cats; gray: not consistent. **(C)** Heatmap of the top 30 consistent differential species across samples.

Species-level differential abundance was first evaluated between domestic cats and *F. bieti* using a threshold of FDR < 0.05 and |log_2_(fold change)| > 1, identifying 866 species significantly more abundant in *F. bieti*, 79 species significantly more abundant in domestic cats, and 5,087 species with no significant difference (Figure 4A). For *O. manul* (*n* = 4), the same FDR-based threshold yielded no significant hits (all FDR > 0.05), likely due to the limited sample size; therefore, to identify robust species-level signatures shared across both wild felid species, we adopted a dual-criteria consistency analysis requiring nominal *p* < 0.05 and |log_2_FC| > 1 in the *O. manul* vs. domestic comparison and FDR < 0.05 and |log_2_FC| > 1 in the *F. bieti* vs. domestic comparison, with the direction of change identical in both comparisons. As shown in Figure 4B, this stringent intersection yielded 31 species consistently enriched in the domestic group, including *Segatella copri, Segatella hominis, Megamonas funiformis, Megasphaera elsdenii* and *Megasphaera stantonii*, and 585 species consistently enriched in the wild felid group, including *Clostridium perfringens, Escherichia coli*, and *Clostridium novyi*, while the remaining 9,082 species showed no consistent difference. A heatmap of the top 30 most abundant species among those exhibiting consistent differential abundance clearly separated domestic from wild samples (Figure 4C).

### 3.4 Identification of differential pathways

**Figure 5:**
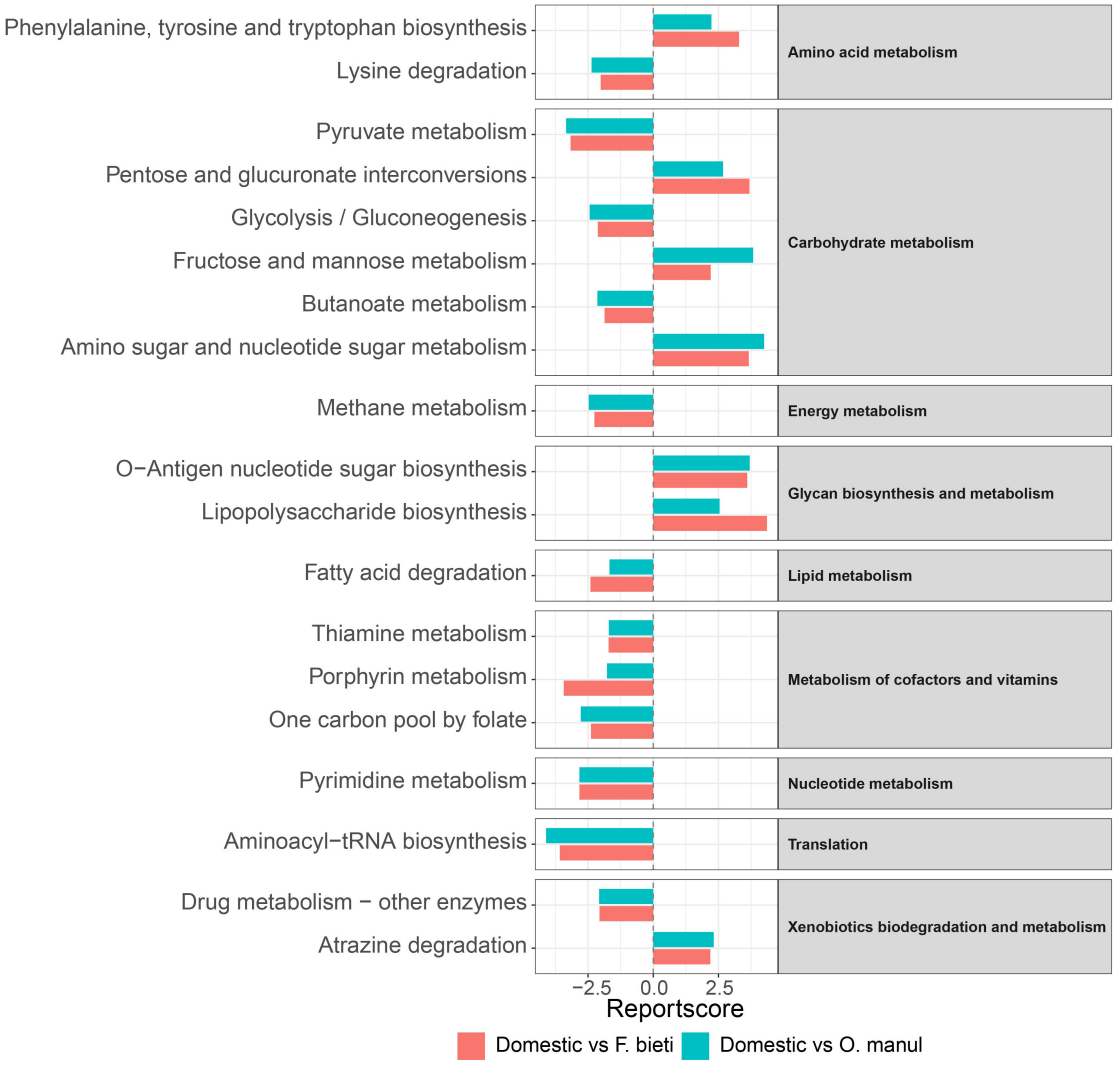
KEGG pathway differences. Positive Reporter Score: enriched in wild felids; negative: enriched in domestic cats.

Based on the Reporter Score method, which statistically integrates all KOs within a pathway to assess its overall change, significant metabolic alterations were identified. We separately compared domestic cats with *F. bieti* and with *O. manul* using the Reporter Score method. This identified 25 and 27 differentially enriched pathways, respectively (Supplementary Figure S5). Pathways with consistent directional changes across both comparisons were considered as robust signatures differentiating domestic from wild felids. As shown in Figure 5, a positive Reporter Score indicated enrichment in wild felids, whereas a negative score indicates enrichment in domestic cats. Among the consistent pathways, 12 were enriched in domestic cats, including lysine degradation, pyruvate metabolism, glycolysis/gluconeogenesis, butanoate metabolism, methane metabolism, fatty acid degradation, thiamine metabolism, porphyrin metabolism, one carbon pool by folate, pyrimidine metabolism, aminoacyl-tRNA biosynthesis, and drug metabolism—other enzymes. Seven pathways were enriched in wild felids: phenylalanine, tyrosine and tryptophan biosynthesis, pentose and glucuronate interconversions, fructose and mannose metabolism, amino sugar and nucleotide sugar metabolism, O-antigen nucleotide sugar biosynthesis, lipopolysaccharide biosynthesis, and atrazine degradation.

To further dissect the functional units underlying the metabolic differences between domestic cats and the combined wild group (comprising *F. bieti* and *O. manul*), we performed Wilcoxon rank-sum tests on all KO (KEGG Orthology) terms within the 19 differentially enriched pathways. Only those KOs that showed consistent directional changes and reached statistical significance (FDR < 0.05) in both comparisons—domestic cats vs. *F. bieti* and domestic cats vs. *O. manul*—were considered as robust signatures. The complete results were presented in Supplementary File 1.

**Figure 6:**
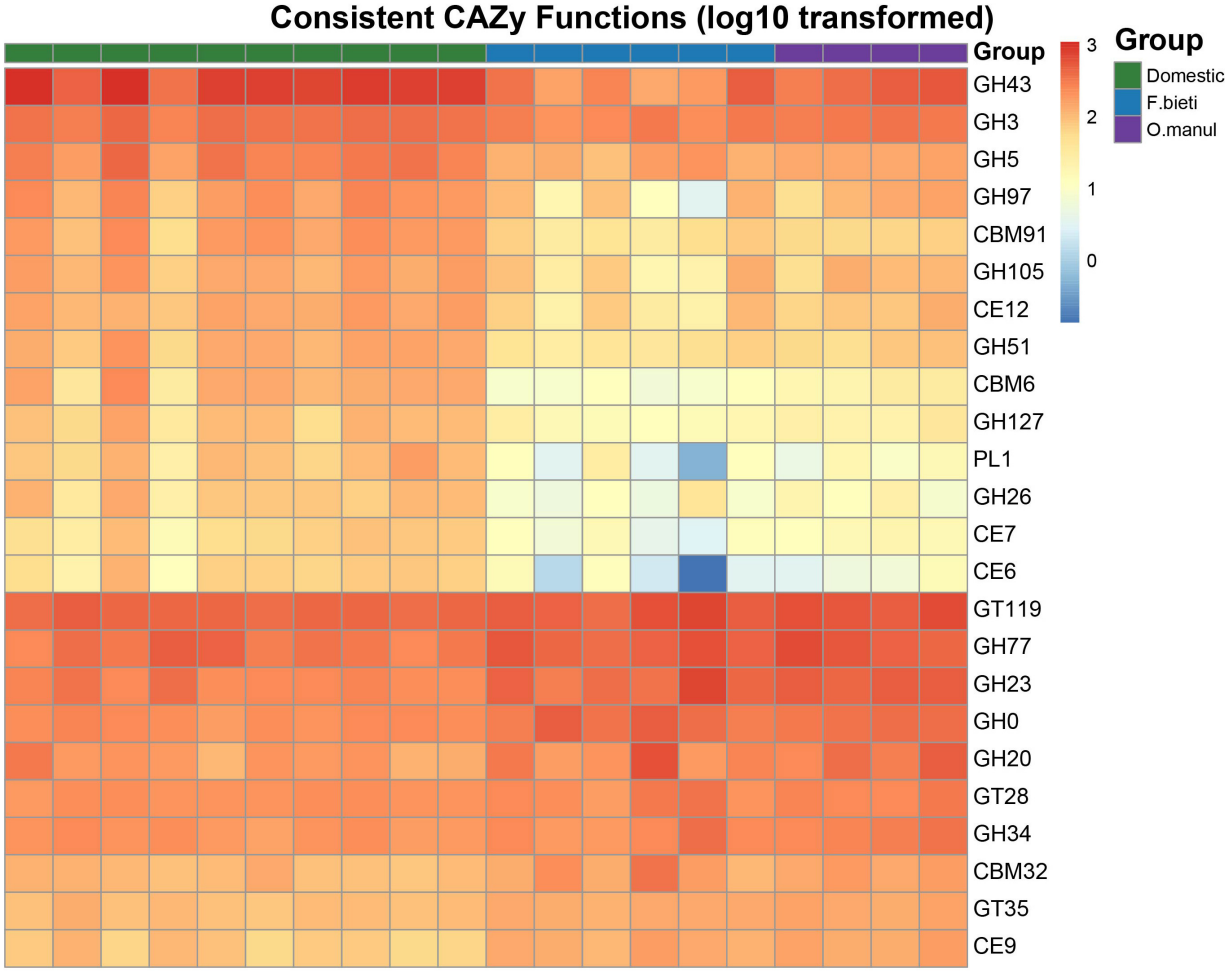
Heatmap of CAZyme family differences.

To further characterize the functional capacity for carbohydrate utilization, we annotated the non-redundant gene catalog against the CAZy database and performed LEfSe analysis (LDA score > 3, Kruskal-Wallis *p* < 0.05) to identify differentially abundant CAZyme families. Comparisons between domestic cats and *F. bieti* yielded 42 differentially enriched families, and between domestic cats and *O. manul* yielded 33 families (Supplementary Figure S6). Families showing consistent directional changes across both comparisons were considered robust signatures differentiating domestic from wild felids. A total of 24 CAZyme families exhibited consistent enrichment patterns: 14 were significantly enriched in domestic cats, including GH43, GH5, GH97, CBM6, CBM91, GH51, GH3, CE12, PL1, GH105, GH127, GH26, CE6, and CE7; 10 were significantly enriched in wild felids, including GH23, GH0, GH77, GT119, GH20, CBM32, CE9, GH34, GT28, and GT35. The relative gene abundances of these 24 consistent CAZyme families were visualized in the heatmap shown in Figure 6.

## 4 Discussion

In this study, the term “domestication” is used as a holistic descriptor that inherently encompasses multiple intertwined factors, including dietary shift (from raw whole prey to processed commercial feed), captive environment (wildlife rescue center vs. controlled research facility), and the genetic background of the host species. It is therefore not possible to completely disentangle the individual contributions of these variables to the observed gut microbiome differences. Furthermore, *O. manul* belongs to the genus Otocolobus, which is a different genus from Felis, the genus that includes both domestic cats and *F. bieti* (Lücht et al., 2019). To minimize the confounding effect of host phylogeny and to identify robust signatures that are shared across wild felids despite their taxonomic divergence, all primary comparisons were performed separately for *O. manul* (*n* = 4) and *F. bieti* (*n* = 6) against the domestic cat group. Only those taxonomic or functional differences that showed consistent directional changes in both wild species were interpreted as potential “wild vs. domestic” signals. Species-specific patterns are reported as such and were not generalized to all wild felids. This analytical strategy provides a more rigorous framework for distinguishing domestication-associated shifts from species-specific idiosyncrasies.

### 4.1 Wild felids exhibit higher gut microbiota diversity

All individuals in the wild group came from rescue operations conducted at the wildlife park. Although prior studies suggest that captivity in zoos may reduce microbial alpha diversity at the species level (Chi et al., 2019), our data demonstrated significantly elevated Chao1 and Shannon indices in the wild group compared to the domestic group. Furthermore, the wild group had a substantially greater number of unique microbial taxa. Collectively, these results indicate that wild felids maintain a gut microbial community with significantly higher richness and evenness than domestic cats. A previous study (Avellán-Llaguno et al., 2025) compared the gut microbiota of three groups: household (D), free-roaming (FR), and free-range domestic (FRD) cats. It found that FRD cats exhibited the highest and most dispersed microbial diversity, while household cats showed lower diversity and a more concentrated community profile. FRD cats also had the highest number of unique species and genera. This aligns with our findings, suggesting that in a free-ranging wild environment, wild felids may promote microbial diversity through exposure to more complex natural habitats and predation on a broader range of food sources.

### 4.2 Domestication reshapes gut microbial composition

This study revealed significant differences in gut microbiota composition among domestic cats, *F. bieti*, and *O. manul* at multiple taxonomic levels. At the phylum level, Bacteroidota was significantly enriched in the domestic group compared to *F. bieti*. Bacteroidota has been associated with a high-carbohydrate diet (Zhang et al., 2025) and is often a dominant phylum in the human gut (Yüksel et al., 2024). This enrichment may therefore be linked to the domestication process and the higher carbohydrate content in commercial cat food. The lack of a significant difference with *O. manul* suggests that the response of Bacteroidota to diet or captivity may be species-specific, or may reflect the smaller sample size of *O. manul* (*n* = 4). Pseudomonadota is ubiquitously distributed in natural environments such as soil and water (Debnath et al., 2025). Its enrichment in the wild group suggests an association with greater environmental exposure. Uroviricota is a major phylum among viral sequences, particularly in microbially active environments such as contaminated sites or high-salinity habitats (Oliver et al., 2024). Its enrichment in the gut of wild felids may be attributed to an increased abundance of bacterial hosts such as Pseudomonadota, which provides a broader range for phage colonization (Zhang et al., 2024).

At the genus level, four genera, including *Segatella*, were identified as characteristic of the domestic group, while eleven genera, including *Clostridium, Fusobacterium* and *Roseburia*, were characteristic of the wild group. In fact, *Segatella*, particularly the species *S. copri*, was the dominant genus with the highest relative abundance in the domestic group. This is consistent with a recent large-scale study in which *Prevotella* (*Segatella*) was identified as a defining genus of one of the two feline gut enterotypes (Deng et al., 2026). In contrast, its relative abundance was less than 1% in the wild group. This differential abundance of *S. copri* was a key contributor to the significant difference observed in the phylum Bacteroidota between the two groups. Different clades of *S. copri* may have overlapping yet potentially complementary capacities for metabolizing dietary carbohydrates, and that the presence of multiple clades may collectively enable efficient breakdown of a broad range of plant polysaccharides (Tett et al., 2019), and soluble fibers present in some commercial cat foods can serve as metabolic substrates for this species (Xiao et al., 2024). In contrast, the natural prey of wild felids is high in protein and low in fiber, creating a gut environment that favors proteolytic bacteria like *Clostridium*, thereby inhibiting the growth of polysaccharide-dependent *Segatella*. *Megasphaera* ferments sugars to produce beneficial metabolites (Reese et al., 2021). These shifts in the microbial community are likely associated with the consumption of commercial diets rich in processed carbohydrates by domestic cats. Furthermore, the enrichment of *Escherichia* in the wild group may indicate greater exposure to environmental pathogens, potentially linked to the consumption of untreated raw food (Reboul et al., 2025).

Notably, *Roseburia*, recognized as a beneficial bacterium that degrades dietary fiber to produce SCFA, was significantly more abundant in wild felids. Both *Roseburia* and *Alistipes* have been implicated in host immunomodulation and anti-inflammatory effects (Levin et al., 2021; Han et al., 2024), potentially aiding wild felids in coping with the more complex pathogen pressures encountered in natural environments.

At the species level, the gut microbial diversity of domestic cats was significantly reduced, with fewer enriched differential species (31 species). This is consistent with their relatively monotonous diet and living environment (Cho et al., 2025). In the gut of wild felids, the enrichment of species such as *Clostridium perfringens* and *Escherichia coli*, which play important roles in protein and fat metabolism (Aizpurua et al., 2025), may be associated with a high-protein diet. However, the enrichment of these potential pathogens (*C. perfringens* and *E. coli*) also suggests that wild felids may face higher health risks, as they can become opportunistic pathogens under certain conditions, leading to intestinal infections or other systemic diseases (Xie et al., 2025). Therefore, the high gut microbial diversity in wild felids may represent a double-edged sword, enhancing their adaptability to complex environments while carrying potential pathological risks.

### 4.3 Domestication reprograms gut microbial metabolism

The most striking finding of this study is the systematic metabolic reprogramming of the gut microbiome during felid domestication. These changes center on carbohydrate utilization, amino acid metabolism, cofactor handling, and cell surface remodeling.

#### 4.3.1 Carbohydrate-active potential and energy metabolism

Wild felids harbored significantly higher abundances of microbial genes encoding pectate disaccharide-lyase (K01731, pentose and glucuronate interconversions) and a glucose phosphotransferase system (PTS) component (K02779, glycolysis/gluconeogenesis), indicating an enhanced capacity to degrade plant cell wall polysaccharides and scavenge monosaccharides from diverse substrates (Xu et al., 2023). Within the butanoate metabolism pathway, wild felids consistently showed enrichment of butyrate kinase (K00929) and the two subunits of butyryl-CoA: acetate CoA-transferase (K01034/K01035), key enzymes that catalyze the final two steps of butyrate production (Singh et al., 2023). Thus, the gut microbiota of wild felids has a higher genetic potential for generating butyrate, a short-chain fatty acid important for gut health.

In domestic cats, the gut metagenome was enriched for hexokinase (K00844) in glycolysis/gluconeogenesis and fructose/mannose metabolism, as well as acetoacetate decarboxylase (K01574) in butanoate metabolism. Acetoacetate decarboxylase converts acetoacetate to acetone, shifting carbon flux away from butyrate and toward ketone body formation. This pattern, together with the lower abundance of butyrate-synthesis genes, suggests that the domestic cat microbiota prioritizes glucose phosphorylation and ketone metabolism over butyrate fermentation (Xu et al., 2023).

The carbohydrate-active enzyme (CAZy) analysis further supported this functional divergence. Domestic cats consistently showed enrichment of 14 CAZyme families: GH43, GH5, GH97, CBM6, CBM91, GH51, GH3, CE12, PL1, GH105, GH127, GH26, CE6, and CE7. Many of these families are involved in the hydrolysis of plant polysaccharides—for example, GH43 (β-xylosidase/α-L-arabinofuranosidase), GH5 and GH26 (mannanases), GH51 (α-L-arabinofuranosidase), GH3 (β-glucosidase), GH97 (α-glucosidase), PL1 (pectate lyase), and the carbohydrate-binding modules CBM6/CBM91 (Chen et al., 2018). These observations indicate an enhanced genetic potential for breaking down arabinoxylans, mannans, pectins, and other soluble fibers typical of commercial extruded diets (Keggi and Doran-Peterson, 2020). In contrast, wild felids consistently exhibited enrichment of 10 CAZyme families: GH23, GH0, GH77, GT119, GH20, CBM32, CE9, GH34, GT28, and GT35. GH23 (lysozyme-type) and GH20 (β-hexosaminidase) may be involved in peptidoglycan turnover or degradation of host-derived glycans (Han et al., 2020), while GH77 (amylomaltase) and the glycosyltransferases GT28/GT35 could reflect a more versatile capacity to process both dietary and bacterial cell wall components (Mareček et al., 2021).

#### 4.3.2 Nitrogen metabolism: from exogenous catabolism to endogenous synthesis

Wild felids showed enrichment of microbial genes for lysine decarboxylase (K01582) and the two subunits of beta-lysine 5,6-aminomutase (K18011, K01844) in the lysine degradation pathway. These genes participate in the conversion of lysine to acetyl-CoA and other intermediates, consistent with a greater reliance on exogenous amino acid catabolism (Berg et al., 2017). Domestic cats, in contrast, displayed higher abundances of chorismate mutase (K06208) and tryptophan synthase alpha chain (K01695)—both part of the phenylalanine, tyrosine and tryptophan biosynthesis pathway. Thus, the domestic cat gut microbiota appears to compensate for the specific amino acid composition of commercial feed by enhancing endogenous synthesis of aromatic amino acids, particularly tryptophan.

#### 4.3.3 Cofactor metabolism: shifting from *de novo* synthesis to salvage pathways

Wild felids consistently showed higher abundances of microbial genes encoding thiamine kinase (K07251) in thiamine metabolism and dihydrofolate reductase (K13938) in the one-carbon pool by folate, both of which are involved in cofactor biosynthesis (Huang et al., 2018; Zhao and Kaldis, 2022; Sehrawat et al., 2024). Domestic cats exhibited enrichment of 5-formyltetrahydrofolate cyclo-ligase (K01934) in the same one-carbon pathway, an enzyme associated with the salvage of folate derivatives. These data indicate a shift from *de novo* synthesis of vitamins and cofactors in wild felids toward greater reliance on salvage pathways in domestic cats.

#### 4.3.4 Cell surface remodeling: adaptation to gut environment

Lipopolysaccharide (LPS) structure influences bacterial survival and host interactions (Di Lorenzo et al., 2021). The consistently significant KO in cell surface remodeling was the (galactosyl) LPS 1,2-glucosyltransferase (K12985) in the LPS biosynthesis pathway, which was enriched in wild felids. This enzyme adds a galactosyl residue to the LPS core, potentially generating more complex and immunostimulatory LPS structures, likely enhancing resilience in a competitive, variable gut ecosystem (Constante et al., 2017; Laplanche et al., 2025).

### 4.4 Limitations

Several limitations should be acknowledged. First, the experimental design does not allow us to disentangle the individual contributions of diet, environment, captivity duration, and host species identity; the term “domestication” as used here captures their combined effect. Second, the sample size is modest (*n* = 20), particularly for *O. manul* (*n* = 4), which limits statistical power for species-stratified analyses and may have prevented detection of some true differences. Third, we did not perform cross-cohort validation using independent public datasets, so the generalizability of our findings to other cat populations remains to be tested. Fourth, metagenome-assembled genomes (MAGs) were not reconstructed in this study, which precludes direct linking of functional genes to specific taxa. Fifth, while we report enrichment of genes encoding specific metabolic pathways, these are potential capacities rather than measured metabolic fluxes; direct evidence would require complementary approaches such as metabolomics or *ex vivo* fermentation assays. Sixth, we did not profile antibiotic resistance genes (ARGs) or virulence factors (VFs) in the current study. The wild animals included here were rescued individuals that had received veterinary medical interventions, including antibiotics, with variable and incompletely documented histories; any ARG or VF signals would therefore be strongly confounded. Such analyses are beyond the scope of this nutrition-focused investigation and should be addressed in future studies using animals without medical interventions. We also note that the study was funded and carried out by a commercial pet food company; therefore, independent validation by non-affiliated research groups is necessary. Despite these limitations, the consistent signatures across two distinct wild felid species and the stringent statistical thresholds support the robustness of the main conclusions.

## 5 Conclusions

This comprehensive metagenomic comparison reveals that domestication profoundly reshapes the gut microbiota of felids, with wild cats (Pallas's cat and Chinese mountain cat) maintaining significantly higher microbial diversity and a larger reservoir of unique taxa than domestic cats. Domestication consistently enriches genera such as *Segatella, Megasphaera*, and *Megamonas*, along with species like *Segatella copri* and *Megamonas funiformis*, while wild felids harbor higher abundances of *Clostridium, Fusobacterium, Roseburia*, and *Escherichia* species. Functionally, the gut metagenome of wild felids exhibits enhanced potential for butyrate production and more complex lipopolysaccharide structures, supporting gut health and resilience in pathogen-rich environments. In contrast, domestic cats show a marked shift toward plant polysaccharide degradation, with consistent enrichment of 14 CAZyme families (e.g., GH43, GH5, PL1, CBM6) that hydrolyze soluble fibers typical of commercial diets, alongside metabolic reprogramming from exogenous amino acid catabolism to endogenous synthesis of aromatic amino acids, and from *de novo* cofactor synthesis to salvage pathways. Notably, while wild felids possess higher diversity, their enrichment of potential pathogens (e.g., *Clostridium perfringens, Escherichia coli*) suggests a trade-off between adaptability and opportunistic infection risks. These findings illuminate how domestication—encompassing diet, environment, and host genetics—has systematically restructured the feline gut ecosystem, providing a crucial metagenomic baseline for feline nutrition, health management, and the evolutionary understanding of host-microbiome coevolution.

## Data Availability

The raw sequence data have been deposited in the NCBI database under accession number PRJNA1430165. The R scripts used for statistical analyses and figure generation have been deposited in a public GitHub repository: https://github.com/wjl120587/felid_microbiome_analysis/.

## Supplementary material

Supplementary Figure S1 Rarefaction curves of 20 samples. Curves approached saturation.

Supplementary Figure S2 PCoA plot of β-diversity among the four breeds of felines.

Supplementary Figure S3 LEfSe results (domestic vs. *O. manul*). Bar plot (LDA > 3) and cladogram (LDA > 4).

Supplementary Figure S4 LEfSe results (domestic vs. *F. bieti*). Bar plot (LDA > 3) and cladogram (LDA > 4).

Supplementary Figure S5 Reporter score for domestic vs. *O. manul* (left) and domestic vs. *F. bieti* (right). Positive: wild-enriched; negative: domestic-enriched.

Supplementary Figure S6 LDA bar plots of CAZy families. Left: domestic vs. *F. bieti*; right: domestic vs. *O. manul*.
